# Natural warming differentiates communities and increases diversity in deep-sea Ridge Flank Hydrothermal Systems

**DOI:** 10.1038/s42003-024-06070-3

**Published:** 2024-03-28

**Authors:** Anne M. Hartwell, Anna E. Wheat, Jennifer A. Dijkstra

**Affiliations:** 1 University of New Hampshire Center for Coastal and Ocean Mapping/Joint Hydrographic Center, 24 Colovos Rd, Durham, NH USA; 2 Oregon State University, 1500 SW Jefferson Ave, Corvallis, OR 97331 USA

**Keywords:** Climate-change ecology, Biooceanography, Biodiversity

## Abstract

Ridge Flank Hydrothermal Systems have discrete pockets of fluid discharge that mimic climate-induced ocean warming. Unlike traditional hydrothermal fluids, those discharged by Ridge Flank Hydrothermal Systems have a chemical composition indistinguishable from background water, enabling evaluation of the effect of warming temperature. Here we link temperature and terrain variables to community composition and biodiversity by combining remotely operated vehicle images of vent and non-vent zone communities with associated environmental variables. We show overall differences in composition, family richness, and biodiversity between zones, though richness and diversity were only significantly greater in vent zones at one location. Temperature was a contributing factor to observed greater biodiversity near vent zones. Overall, our results suggest that warming in the deep sea will affect species composition and diversity. However, due to the diverse outcomes projected for ocean warming, additional research is necessary to forecast the impacts of ocean warming on deep-sea ecosystems.

## Abstract (web summary)

A study of opportunity on deep-sea Ridge Flank Hydrothermal Systems, which host low-temperature, chemically insignificant venting, suggests that warmer temperatures are a contributing factor to changing community composition and higher biodiversity.

## Introduction

Rising temperatures are leading to the restructuring of ecological communities, as species adjust to changes in air and ocean temperatures^1,2^. However, the impacts of global warming vary across ecosystems and are layered over the variable patterns of biodiversity^3,4^. Species that benefit from warming tend to thrive and expand their geographic ranges^5–7^. Conversely, species that are more limited in their thermal preferences may decline as individuals perish or relocate to more suitable areas^8,9^. In a recent study, the influence of rising temperatures was more conspicuous in marine ecosystems compared to terrestrial ones, with cooler locations experiencing an increase in biodiversity as temperatures rise^10^. Cooler regions are also expected to have faster colonization and slower extinction rates^11^. Though a few studies^12–16^ have examined the relationship between ocean warming and diversity and species composition in the deep sea (>1-kilometer), Ridge Flank Hydrothermal Systems (RFHS), a low temperature fluid venting system, may provide additional insight into the influence of warming on deep-sea communities.

**Fig. 1:**
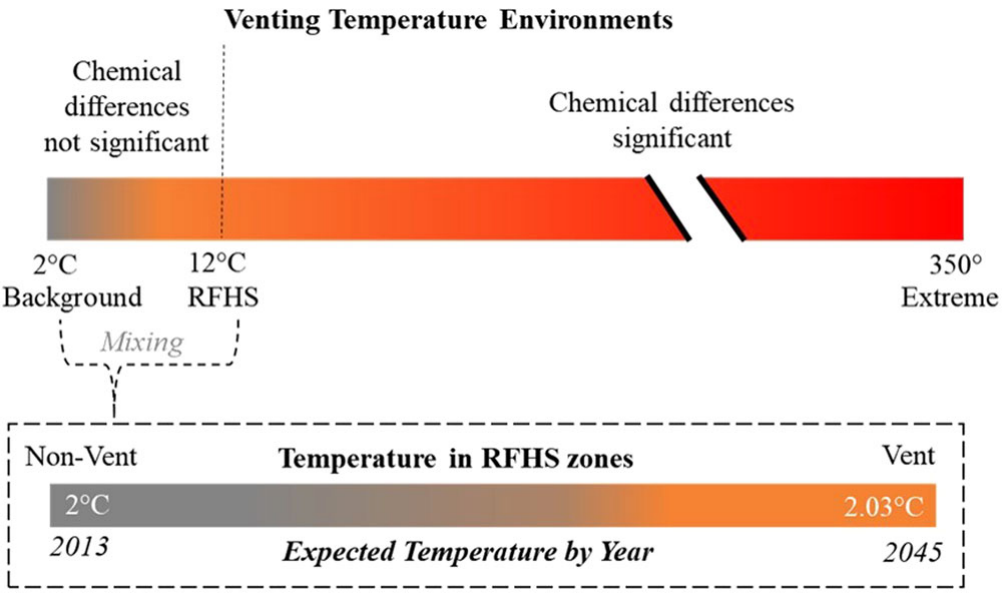
Schematic representation of RFHS venting temperatures relative to hydrothermal vents and expected ocean warming. Illustration of vent and non-vent zones. The top color bar is the range of venting temperatures in the deep sea. The dotted lines mark temperature cutoff for which there are associated chemical differences or not. The bottom color bar is the range of temperatures measured 6-meter off the bottom at RFHS over vent and non-vent zones, and below the bar, the corresponding years those temperatures are expected to be reached in some parts of the deep Pacific. The temperature range above the vent and non-vent zones is on the same order of magnitude as the temperature change expected in some regions if warming increases at a low estimate rate of 0.01 °C per decade, as has been recorded in the past and is expected to continue into the future^19,34^.

At RFHS, cold bottom water enters the crust, flows laterally while being warmed from adiabatic heating until a pressure differential drives fluid from the crust at outcroppings along the ridge flank where it diffuses into bottom water^17^. RFHS are different from hydrothermal vents, which discharge fluids with extreme temperatures (100’s °C) and toxic and heavy metals. First, the seawater from the fluids released at the venting sites are not chemically altered, i.e., the major ion water chemistry is indistinguishable from the surrounding water (water emissions at RFHS do not contain toxic chemicals like hydrogen sulfide)^18^. Second, though temperature is above ambient in discharged fluids, it is still on the same order of magnitude (10 °C) as the surrounding water (1.8 °C)^18^. Important in these systems is that the warm venting fluid quickly mixes with colder background water and results in a measured temperature anomaly in the zones surrounding a venting site (Fig. 1) that is on the same order of magnitude (tenths of a degree) as temperature increases predicted from climate change described by Purkey and Johnson (2010)^19^.

In general, the deep sea is considered to have little temperature variation and species inhabiting thermally stable environments tend to be more stenothermal than those that inhabit more thermally variable environments. Studies suggest that in these environmentally stable systems (e.g., polar or deep-sea environments), small changes in temperature can lead to changes in species composition and diversity^6,20^ as temperature effects physiological processes crucial for growth and reproduction and species settlement^6,13,21^.

Like other benthic ecosystems, diversity in the deep sea is patchy. To date, diversity has been examined on several different seafloor features including seamounts, canyons, seeps, hydrothermal vents and on the abyssal plain. Hard substrate is critical for the settlement of sessile megafauna as it provides a surface for attachment. Many of these sessile species are foundation species (i.e., corals and sponges) that enhance local biodiversity by creating additional sheltered areas and secondary substrate that extend into nutrient-rich bottom currents^22–25^. Seamounts, outcrops, and canyons, therefore, serve as diversity hotspots due to the abundant resources of greater food availability and surface area^23,26^. Hydrothermal vents represent specialized deep-sea environments where diversity is low and biomass is high^27,28^. This is due to the venting fluids that release chemically altered seawater and the surrounding organisms rely on symbiotic relationships with chemosynthetic microbes for growth and survival^29–31^. The broader area surrounding vents, those that are transitioning from extreme conditions towards ambient ones, tends to be an area that is intermediate between the extreme conditions of hydrothermal vents and the wider deep-sea region^32^. The species in these transition zones are cosmopolitan and lack symbionts but can show behavioral, physiological or biochemical adaptations that help them tolerate reduced concentrations of vent fluid toxicity^33^.

Ocean warming in the deep sea has been observed in past decades and is predicted to continue with the changing climate^19,34^. In the Pacific Ocean, evolving patterns of water-mass circulation related to shifting winds drive warming in water depths greater than 3-kilometers at rates of 0.01–0.1 °C per decade^19^. In addition to warming, dissolved oxygen (DO) has declined over the past few decades and this trend is predicted to continue for decades to come^35,36^. In the deep sea, DO decline is driven by changes in ocean circulation and ventilation^35,36^, with negligible influence from temperature and salinity^36^. As environmental and seafloor variables can alter the spatial distribution of species^6,8,37–39^, changes to diversity and species composition in areas that have pockets of warmer water, such as RFHS, should be explored.

As of 2022, only two RFHS were known, Dorado outcrop and outcrops in the Davidson Seamount Management Zone Monterey Bay National Marine Sanctuary (DSMZ-MBNMS). A third site near Dorado outcrop was discovered in June of 2023^40^. Here we describe the composition and diversity of taxa in vent and non-vent zones on Dorado outcrop and DSMZ-MBNMS. By documenting taxa that occur on these RFHS, we provide a baseline that can be used to determine temporal patterns of community change and for the management of these systems^9,20^. Although only three RFHS have been discovered, many more are likely to exist. There is an estimated 1-10 million seamounts 100-meters or taller on the seafloor^41,42^, and those on ridge flanks could facilitate venting^43^. We then examined the relationship between environmental seafloor variables, species composition and diversity among vent and non-vent zones.

Results of our study show differences in community composition and structure between vent and non-vent zones. Vent zones displayed higher diversity, but this increase was statistically significant only at DSMZ-MBNMS. This could be attributed to the larger temperature disparities observed between vent and non-vent zones at this specific location. Furthermore, temperature exhibited a positive relationship with both diversity and richness, with a notably stronger correlation at DSMZ-MBNMS compared to Dorado. Our results suggest that temperature may play a role in enhancing biodiversity and supporting foundation species in RFHS. However, further investigation is necessary to fully grasp the broad-scale impacts of ocean warming on deep-sea environments.

## Results

### Community Composition

**Fig. 2:**
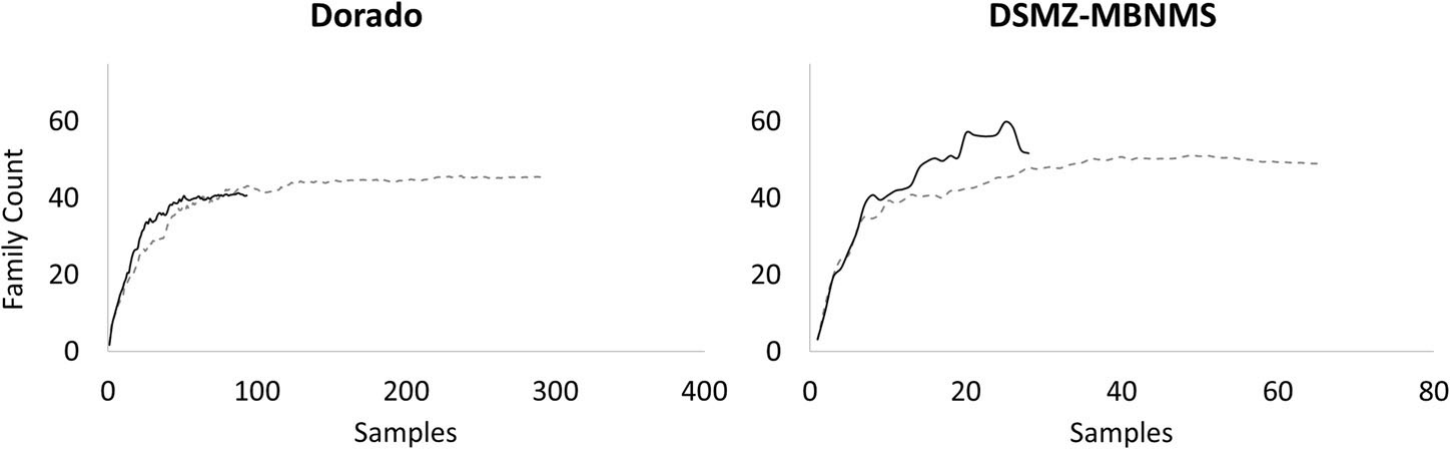
Family accumulation curves. An estimation of family richness at Dorado and DSMZ-MBNMS. The value plotted is a bias-corrected estimate of the family richness for a subsample taken over 100 randomizations using Choa2 prediction. Dashed grey lines are vents; solid black lines are non-vents. For Dorado, *n* = 292 for vent zones and *n* = 92 for non-vent zones. For DSMZ-MBNMS, *n* = 64 for vent zones and n = 27 for non-vent zones.

Asymptotic rarefaction curves indicate that a sufficient number of families were captured in vent and non-vent zones on Dorado and DSMZ-MBNMS to represent the community (Fig. 2)^44^. Overall, family richness was greater in vents at both Dorado and DSMZ-MBNMS (vent: 45 ± 4, non-vent: 40 ± 7; vent: 49 ± 3, non-vent: 47 ± 11, respectively). However, family richness and diversity (Shannon Diversity Index) between vent and non-vent zones were only significantly different at DSMZ-MBNMS (*p* = 0.0005, *p* = 0.0035, respectively; diversity indices reported in Supplementary Table 1 and Supplementary Table 2). Evenness showed no difference between vent and non-vent zones at either Dorado or DSMZ-MBNMS.

**Fig. 3:**
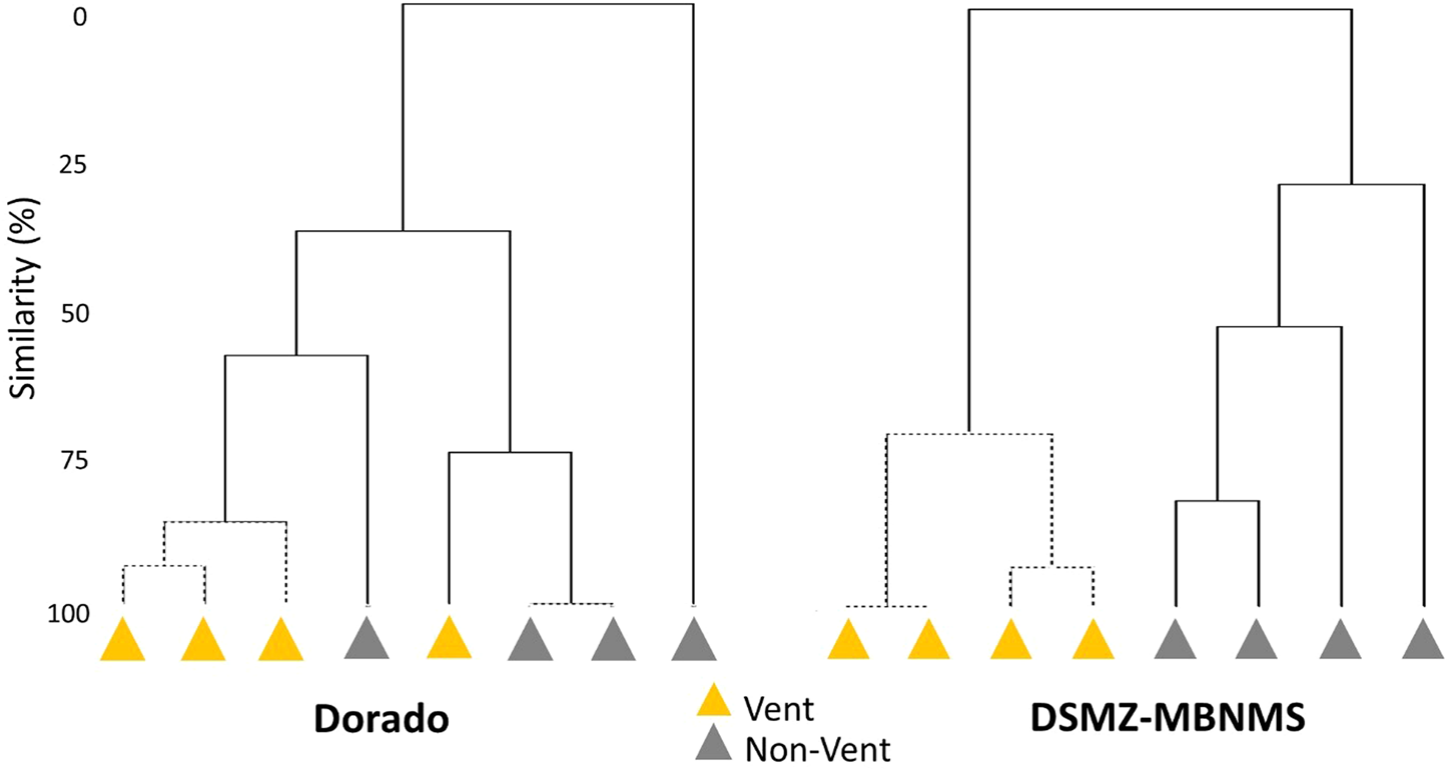
Dendrogram of zones. Cluster-Analysis dendrogram of zones for both Dorado and DSMZ-MBNMS. Similarity index: Bray-Curtis, transformation: square root; *p* = 0.05; samples connected by dotted lines: no significant differences; cluster mode: group average. For both Dorado and DSMZ-MBNMS the number of vent and non-vent zones is *n* = 8 (total, 4 for each zone type).

The families display clearer grouping of zone types at DSMZ-MBNMS compared to Dorado Outcrop (Fig. 3).

**Fig. 4:**
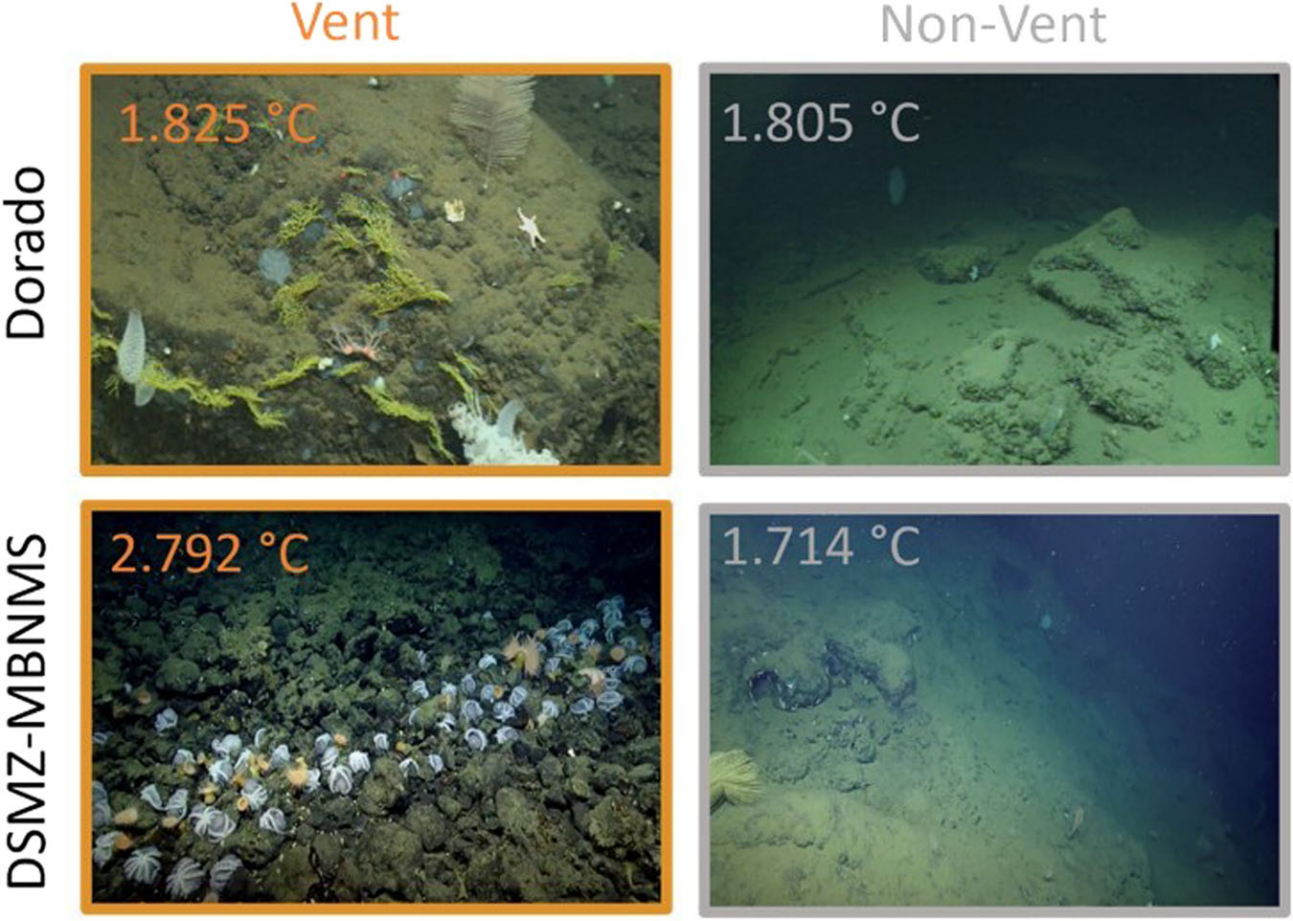
Visual comparison of vent and non-vent zones on RFHS. Remotely operated vehicle (ROV) stills displaying the contrast between vent and non-vent zones at Dorado and DSMZ-MBNMS. Water temperatures measured with probes on the ROV are shown in the top left corner of each image. Images are from the Jason Virtual Van^73^ and the Nautilus Live Dive Recordings YouTube channel^74^ for Dorado and DSMZ-MBNMS, respectively.

There were significant differences in family composition between vent and non-vent zones at both Dorado (*p* = 0.028) and DSMZ-MBNMS (*p* = «0.05) (Fig. 4). T scores indicate the strength of the difference among vent and non-vent zones. The larger negative T score at DSMZ-MBNMS (*T* = −13.31) indicate vent and non-vent zone communities are more dissimilar than vent and non-vent communities at Dorado (*T* = −2.40).

The glass sponge family Euplectellidae (Indicator value (IV) = 20.6; *p* = 0.0002), brooding octopus *Muusoctopus sp*. (IV = 5.6; *p* = 0.0002), and the sea star family Goniasteridae (IV = 2.4; *p* = 0.0176) were the strongest indicators of vent zone communities at Dorado. Indicator families of non-vent zones at Dorado were Brisingidae sea stars (IV = 5.5; *p* = 0.0050), Porifera sp. 1 (IV = 4.1; *p* = 0.0496) and sp. 2 (IV = 1.7; *p* = 0.0324), non-brooding *Muusoctopus sp*. (IV = 1.5; *p* = 0.0236), and white squat lobster family Munidopsidae (IV = 1.1; *p* = 0.0390). At DSMZ-MBNMS, the brooding *Muusoctopus sp*. (IV = 44.4; *p* = 0.0002) and a Brisingidae sea star (IV = 33.7; *p* = 0.0016) were indicators of vent communities. Non-vent zone indicators were sea stars from the families Goniasteridae (IV = 25.8; *p* = 0.0020) and Zorasteridae (IV = 42.4; *p* = 0.0020), two unidentified anthozoans (sp.1 IV = 14.3; *p* = 0.0062; sp. 2 IV = 10.7; *p* = 0.0260), a glass sponge from the family Rossellidae (IV = 10.7; *p* = 0.0290), an unidentified sponge from the family Demospongiae (IV = 17.9; *p* = 0.0022), and a Munidopsidae white squat lobster (IV = 25.0; *p* = 0.0318). Indicator values at DSMZ-MBNMS were at least twice the values at Dorado, indicating that families found in a specific zone have limited association with the other zone. Community composition tables for both study sites are available as Supplementary Data 1 and Supplementary Data 2.

### Environmental Variables

**Fig. 5:**
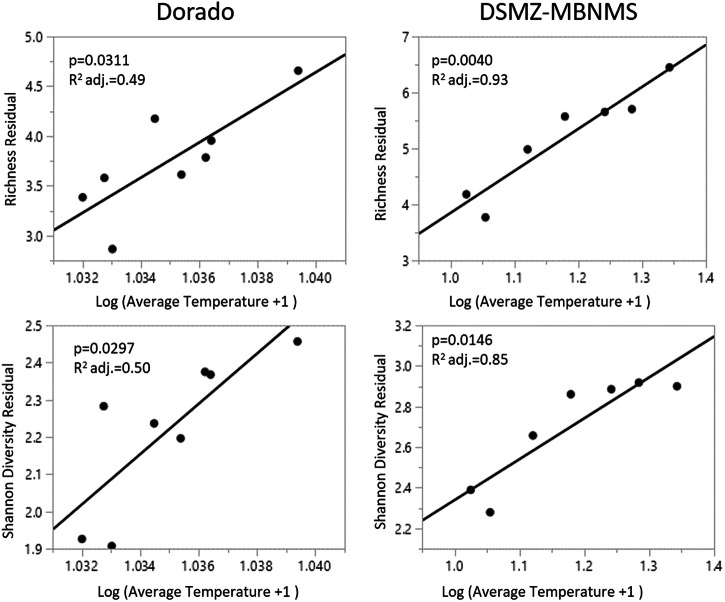
Least squared multiple regression of diversity indices verse temperature. Standard least squared regression of family richness and Shannon diversity against average temperature at Dorado and DSMZ-MBNMS. Family richness and diversity increased with temperature. For Dorado, *n* = 8 values for each zone, and for DSMZ-MBNMS *n* = 7, for each zone, excluding the outlier.

The only environmental variables that were significantly different (95% confidence interval unless otherwise stated) at vent and non-vent zones were geomorphic setting (*p* = 0.0165) and mean temperature (*p* = 0.0202) at Dorado, and northerness (p = 0.0365) and mean temperature (*p* = 0.0003) at DSMZ-MBNMS. Mean temperature was the only significant term in the standard least squared multiple regression for family richness at Dorado Outcrop (adj. R^2^: 0.49, *p* = 0.0311) and DSMZ-MBNMS (adj. R^2^: 0.93, *p* = 0.0040) and Shannon Diversity Index at Dorado (adj. R^2^:0.50, *p* = 0.0297) and DSMZ-MBNMS (adj. R^2^:0.85, *p* = 0.0146) (Fig. 5). No other relationships between abundance, diversity, or evenness and environmental variables were found.

BEST analysis of all environmental factors, including DO, that correlated most strongly to family composition of Dorado Outcrop vent communities was temperature (rho = 0.098, *p* = 0.002) and of non-vent communities was geomorphic setting (rho = 0.066, *p* = 0.038). At DSMS-NMS, temperature and northerness significantly correlated with non-vent family composition (rho = 0.407, *p* = 0.002) and no relationship was observed for vent family composition.

## Discussion

The deep sea is characterized by relatively stable environmental conditions in which small changes in temperature may alter community or family distribution patterns^6,20,45^. This study of low-temperature hydrothermal environments represents an opportunity to further our understanding of deep-sea community responses to warmer water temperatures. Our results indicate that community composition is different between vent and non-vent zones. At vent zones, discharge fluid (10–12 °C at source) quickly mixes with the ambient temperature to provide a temperature gradient across the vent zone in which temperature declines with distance from the source. This temperature gradient may provide a habitat for a wide variety of families, as observed in this study, with various thermal affinities or those that have wide thermal tolerances (e.g., Euplectellidae, Davison et al., 2019^46^), facilitating diversity at these sites. This is particularly noticeable at DSMZ-MBNMS where distinctive communities were observed between vent and non-vent zones, and temperature explained a higher percentage of the variance in richness and diversity than at Dorado. This may be due to the greater observed temperature differences between vent and non-vent zones at DSMZ-MBNMS, although more research is required. Though temperature can negatively influence species distribution^9^, it can also exert a positive influence on richness. Indeed, a recent study in the North Atlantic revealed a positive correlation between richness and temperature and attributed this correlation to the colonization of new community members and sustained existence of established members, notably foundation species with larval development^11^.

The presence of octopus in vent zones at both Dorado and DZMS^47,48^, may support greater diversity within vent zones. Dense aggregations of brooding octopus are observed to cluster around vent sites, likely because warmer temperatures increase the rate of embryonic development^47^. Octopus lay their eggs close to a venting site and brood them while their bodies undergo senescence. Both the egg clutches and senescing octopuses are a food source that may attract predators^49^. Further, the remains of the senesced octopus may enhance local concentrations of detritus that is entrained in the water column and made available to many sessile filter feeders^13,47,50^. A potential increase in predators and higher food concentrations may also directly increase diversity in vent zones. However, given that brooding octopus surround vent sites, the increase in species richness at these sites is likely a direct result of temperature that facilitates the presence of brooding octopus and sessile filter feeders.

Overall differences in family richness and composition between the two RFHS may also be related to connectivity, as families that occur on RFHS appear to be the same as those that inhabit adjacent non-RFHS, suggesting larval exchange between these two habitats^51^. This principle extends to the apparent overall differences in family composition at Dorado and DSMZ-MBNMS. The greater larval supply from surrounding outcrops and the Davidson Seamount may increase the chance of a species’ successful establishment. For example, deep-sea anemones have limited dispersal as their larvae may be lecithotrophic and they have crawl away planula^52,53^, which may explain the high frequency of their occurrence at DSMZ-MBNMS. Anemones were rarely observed at Dorado and their life-history characteristics may limit their expansion at this location. Geology may also contribute to the differences in family composition between DSMZ-MBNMS and Dorado. Differing formations may result in vastly different terrain, which could affect the surrounding oceanography (e.g., intensity of ocean currents) that control larval dispersal, nutrient availability and other variables that regulate species distribution. However, to fully understand how geologic history affects community composition or family richness, additional sampling on RFHS is required.

This study suggests that warming will either directly or indirectly influences species composition and potentially increase diversity. More research is necessary to understand the generality of these results as temperatures are not uniform across the deep sea^19,34,54^. Additionally, other factors such as food availability, dispersal/hydrodynamics, biotic interactions, and suitable habitat - all of which are affected by ocean warming - differ from one region to another. Consequently, regions will experience changes in these factors to varying degrees, with certain regions experiencing more or less impact from one factor compared to another^4,6,37,55–59^. For example, the unequal availability of plankton that sinks to the seafloor as detritus (i.e., zooplankton, plankton) will drive non-uniform changes to deep-sea communities as they depend on the sinking of detritus^22,60^. An increase in detritus can drive greater abundance of deep-sea species by inhibiting distribution^61^ and a dearth can reduce the abundance of organisms in the deep sea^62,63^. Other factors such as climate driven changes to dispersion and circulation can introduce new species to a system, leading to species turnover^6,54,64^.

There may also be species- or family-specific responses to ocean warming, which presents challenges when predicting community response to warming. The effect of temperature on biological processes is not static across biological scales (cells to populations)^4,6,39^, in which one biological process may benefit (e.g., larval development) but another may suffer (e.g., metabolic processes). At the source of venting, the elevated temperature is likely beyond the thermal tolerance of some deep-sea organisms, while others may be more tolerant of greater temperatures^6^. These species-specific thermal tolerances will have effects on space occupancy and competition and predation, factors that will alter species interactions and their distribution at RFHS. A further additional layer of complexity in expanding our observations is that the communities observed at DSMZ-MBNMS and Dorado are different with many more individuals and families observed at DSMZ-MBNMS, making the direction of community responses to ocean warming difficult to predict. Distances among the network of interacting community members are closer at DSMZ-MBNMS than at Dorado as the surrounding outcrops at DSMZ-MBNMS are closer. These same surrounding outcrops will also be influenced by gains/losses of species or families depending on timing, resource needs, and interactions, further complicating predictions of ocean warming.

Environmental factors, other than temperature, will vary with ocean warming. For example, DO is influenced by ocean warming via air-sea interactions and mixing/stratification, and the intensity of the impact varies regionally and by depth^36,65^. Oceanographic models predict that the oxygen minimum zone in the Pacific Ocean is predicted to expand^66^, which will have implications for the distribution and abundance of species as low DO concentrations can lead to disruptions in physiological processes (e.g., metabolic pathways). Food concentrations are also not uniform across regions, with some regions experiencing more variability than others with climate change, which will undoubtedly affect reproduction and dispersal of species. Though ocean warming and other climate related changes will not be uniform across regions, our results suggest that ocean warming has the potential to increase diversity.

RFHS provide a natural environment to examine the relationships between temperature and community structure and composition because of changes predicted in climate scenarios. While the direction of family richness as a function of temperature and suspected carbon supply was similar between RFHS, the observed differences in diversity patterns between them revealed that community dynamics, connectivity, and geology are important considerations when exploring the resilience and the response of a system to warming. The results from this study will help to inform predictive models of biodiversity and resource management decisions, particularly with on-going and increasing pressures for deep-sea mining and fisheries.

## Methods

**Fig. 6:**
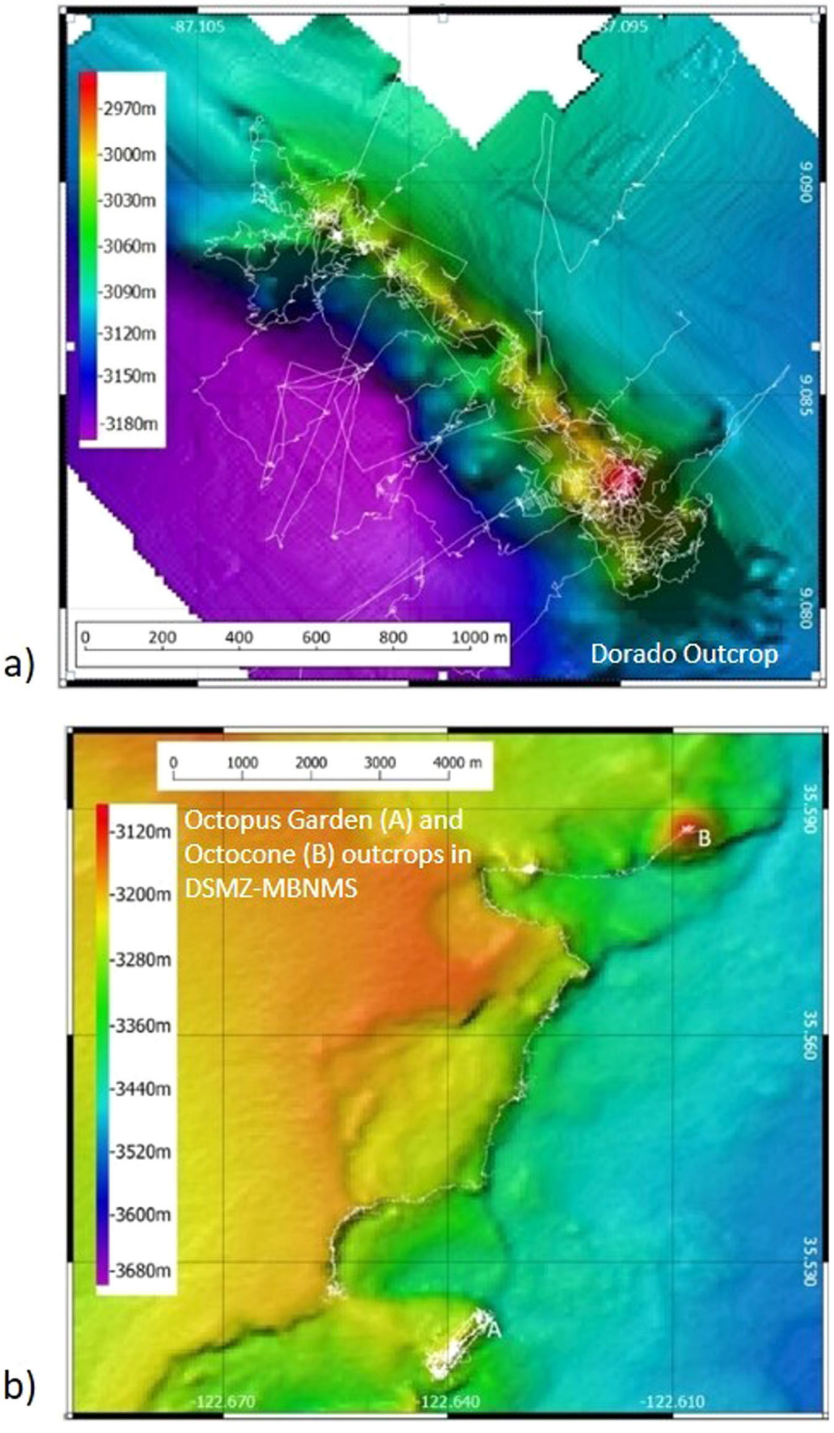
Study site locations. Bathymetric terrain models and ROV dive tracks of RFHS study sites. Color is by depth, white lines are ROV dive tracks. **a** Dorado Outcrop. **b** Outcrops in DSMZ-MBNMS, Octopus Garden (A) and Octocone (B). Maps in the figure were generated using QGIS 3.0.3 and then combined into a single figure using Microsoft PowerPoint by A.M.H. The bathymetric terrain model for Dorado Outcrop was gridded in Fledermaus 7.0 from XYZ data available from the University of Alaska Fairbanks (2013)^67^ on National Centers for Environmental Information. The bathymetric terrain model for DSMZ-MBNMS is available from Marine Geoscience Data System, made available by Caress and Paduan (2023)^68^. Colorbars were generated using Fledermaus 7.0.

This study examines diversity and community composition between vent and non-vent zones from two RFHS locations, Dorado outcrop and DSMZ-MBNMS (Fig. 6; Table 1). The DSMZ-MBNMS has several outcrops, two of which were analyzed for this study. They are approximately 8-kilometers apart and, due to the small distance between the sites, are considered as one location. Both Dorado and DSMZ-MBNMS have cyclic active low-temperature hydrothermal discharge.

**Table 1:** Details for RFHS study sites

Outcrop	Plate	Age of Crust (Ma)	T_max_ of vent fluid (°C)	DO_min_ (µmol)	Depth (m)	Latitude (DD N)	Longitude (DD W)	Height (m)	Area (km x km)
Dorado	Cocos	18–23	12 °C	62	2970	9.08	87.09	150	1 x .5
DSMZ-MBNMS	Pacific	9.8–14.8	10 °C	58	3120	A: 35.56	A: 122.60	A: 150	A: 1 x .3
						B: 35.51	A: 122.63	B: 125	B: 1 x .8

RFHS details for Dorado outcrop and the outcrops in DSMZ-MBNMS, Octopus Garden (A) and Octocone (B). Max Temperature (T_max)_, Dissolved Oxygen minimum (DO_min)_, and Depth are presented as summary values for the DSMZ-MBNMS RFHS.

Dorado and DSMZ-MBNMS were explored using Remotely Operated Vehicles (ROV) and autonomous underwater vehicles (AUV) equipped with camera systems, temperature, and oxygen probes. Ninety-six hours of underwater video footage and still images at Dorado were collected in 2013 (AT26-09) using the ROV Jason deployed from R/V Atlantis and captured a total dive track of ~62-kilometers. Cameras captured three angles: forward, peripheral right, and peripheral left. Fifty-seven hours of underwater video footage and still images at DSMZ-MBNMS were collected in 2019 (NA-117) using the ROV Hercules deployed from the E/V Nautilus for a total dive track of ~127-kilometers. Temperature at Dorado was collected with ROV Jason II and at DSMZ-MBNMS was measured with a probe attached to the ROV Hercules. The large spatial coverage of observations and temperature data collected at outcrops on RFHS enables a comparison of community composition inside and outside of vent zones.

Geomorphological setting was derived from cleaned and quality controlled bathymetric data collected with multibeam echosounders (MBES) by AUV Sentry at Dorado^67^ and the MBARI Mapping AUVs at DSMZ-MBNMS^68^. They were processed and gridded into 1-meter digital terrain models with MB-System software (Supplementary Table 3). Geomorphons were extracted from bathymetric grids using BRESS (Bathymetric and Reflectivity Based Approach for Seafloor Segmentation; Masetti et al., 2018)^69^. The BRESS analytical approach implements principles of topographic openness, pattern recognition, and texture classification to identify a collection of homogeneous and non-overlapping seafloor segments of consistent morphology. We used six geomorphon classifications: flat, ridge, slope, valley, shoulder, and footslope. Except for slope, input parameters were determined by groundtruthing output geomorphons to the input grid and visual observations to validate that the automated classification output matched the bathymetric grid. Slope was extracted in Fledermaus7. The same input parameters were used for both Dorado and DSMZ-MBNMS grids: openness angle of 9 degrees, a search inner/outer radius of 15/45 nodes from the center. Terrain variables were extracted using the TASSE toolbox^70^ as these variables (rugosity, topographic position index, northerness, easterness, and slope, described in Supplementary Table 4) were shown to explain 70% of texture of a seafloor feature, while also reducing redundancy, covariation, and ambiguity in analysis^70^. Variables used in this analysis included northerness, easterness, and topographic position index. Slope from TASSE was excluded to avoid redundancy from it being an input valuable for geomorph extraction, rugosity was excluded to avoid mischaracterizing hydrographic survey artifacts as rugosity, and depth was excluded from analysis because of the varying altitude of the ROV.

### Comparison of Vent and Non-Vent zones

**Fig. 7:**
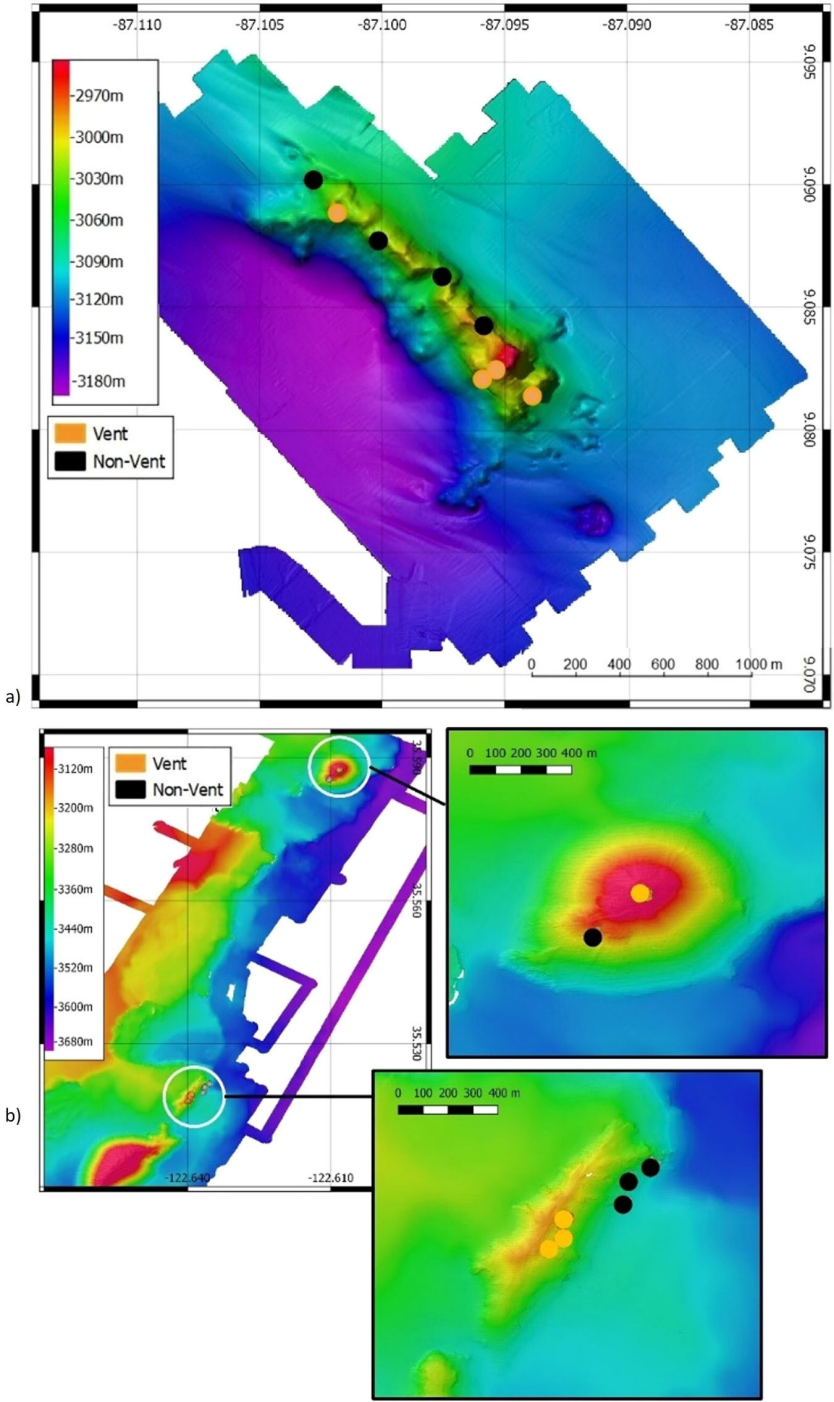
Vent and non-vent zone locations within study sites*.* Location of analysis zones within study sites. **a** Location of vent (orange) and non-vent zones (black) at Dorado. Zone symbols are not to scale. **b** Location of vent (orange) and non-vent zones (black) at DSMZ-MBNMS. Zone symbols are not to scale. All maps in the figure were generated using QGIS 3.0.3 and then combined into a single figure using Microsoft PowerPoint by A.M.H. The bathymetric terrain model for Dorado Outcrop was gridded in Fledermaus 7.0 from XYZ data available from the University of Alaska Fairbanks (2013)^67^ on National Centers for Environmental Information). The bathymetric terrain model for DSMZ-MBNMS is available from Marine Geoscience Data System, made available by Caress and Paduan (2023)^68^. Colorbars were generated using Fledermaus 7.0.

Within each location, we analyzed communities within a 34-meter radius around each vent and non-vent zone; there are four vents and non-vent per RFHS. The area around each vent zone was bound by thermal anomalies greater than 0.01 °C above background temperatures as measured by the AUV probe 6-meters from the outcrop where active venting was observed at Dorado. The same size area was used at DSMZ-MBNMS for consistency and comparison with Dorado. Four was the number of vents selected because that was the total number of vents observed at Dorado in 2013. Areas around non-vent zones were selected using the following four criteria: (1) area of vent and non-vent zones did not overlap, (2) environmental data was available (3) megafauna were present, and (4) geomorphological characteristics were like vent zones (Fig. 7)

Community composition within each zone was determined from still images and underwater video footage collected at Dorado and DSMZ-MBNMS^71,72^. Images were captured at 1-minute intervals and stored on the Jason Virtual Van (JVV; “Jason Virtual Control Van,” 2013)^73^ online database (Dorado) or accessed via YouTube (DSMZ-MBNMS; “Nautilus Live Dive Recordings: NA117 video 82-154,” 2019)^74^. Timestamps and location details were available in JVV for Dorado images. Locations in the DSMZ-MBNMS footage were determined by matching the timestamps of the observation with the timestamps for ROV navigation. Individuals that appeared in overlapping images were only counted once. Though altitude of the ROVs at both RFHS varied along the dive tracks, distance to the seafloor was generally maintained for suitable taxonomic and substrate identification. Megafauna families were identified with the aid of online identification guides: SOEST-HURL^75^, NOAA OER Benthic Deepwater Animal Identification Guide V3^76^, and MBARI deep-sea guide^77^. Consistency in identifications was maintained by generating a catalog for each RFHS and limiting taxa identification to one person at Dorado and two people at DSMZ-MBNMS. Identifications were limited to the family level as they were based on imagery alone. In the deep sea, identification to the family level results in no loss of information about community structure^78^. An exception was for *Muusoctopus sp*., which was classified as brooding or non-brooding because of the significance of brooding to reproductive behavior (i.e., brooding individuals are essentially sessile and non-brooding are wandering).

Numerical environmental data and categorical representation of substrate and geomorph that were converted to numerical values were spatially joined with a grid in QGIS 3.0.3^79–81^. The grid values for each terrain variable within a zone were averaged to compare relative differences between zones. BRESS geomorphons were categorized between 1-6 (Supplementary Table 5). Each grid cell within a zone is assigned an arbitrary numerical value to represent the overlapping geomorph. Substrate was categorized as 0–15 based on the visual observation of bottom type in the ROV footage using the same classification scheme as Wheat et al. (2019)^82^ (Supplementary Table 6). Briefly, for both Dorado and the outcrops in DSMZ-MBNMS, ROV images were analyzed and categorized based on visual appearance of bottom substrate, and then a numerical value was assigned. DO was not included because of its inherent inverse relationship with temperature observed in the discharged fluid^82^ (A.M.H. personal observation DSMZ-MBNMS). Because temperature and DO have such a strong linear correlation, including them both in the regression would weaken the effect of both variables on community differences. We acknowledge that DO is an important factor in deep-sea community distribution and low-oxygen concentrations have an impact on nutrient cycling and are stress factor.

### Statistics and Reproducibility

Rarefaction curves were used to assess if sampling captured a representative number of families at each location for both vent and non-vent zones and to assess family richness in vent and non-vent zones. EstimateS was used to standardize for sample size^83^. Within each individual zone (i.e. determining values for each of the eight zones), richness, evenness, and Shannon Diversity Index were determined using the DIVERSE function in PRIMERv6 (Plymouth Routine in Multivariate Ecological Research). T-test (one way) was used to determine differences in richness, Pielou’s evenness, and Shannon’s diversity between vent and non-vent zones using JMP16.

After calculation of diversity indices, rare species were removed conservatively to reduce noise as described in McCune et al. (2002)^84^, <5% of families were removed as rare species. The value ‘n’ is the cut-off for the minimum number of observations that a family must have to not be considered rare. There were 75 and 90 families at Dorado and DSMZ-MBNMS, respectively, so we used a conservative value of *n* = 3 at both RFHS.

Grouping of zone samples was visualized with a cluster analysis in PRIMERv6. Multiple Response Permutation Procedure (MRPP) was used to detect differences between vent and non-vent communities within Dorado and DSMZ-MBNMS. MRPP calculates the probability that the weighted average distance between samples within vent (*n* = 293 Dorado, *n* = 56 DSMZ-MBNMS) or non-vent zones (*n* = 95 Dorado, *n* = 27 DSMZ-MBNMS) will be significantly different than the weighted average distance of randomized samples^85,86^. MRPP analysis was calculated using a Bray-Curtis distance matrix on presence/absence data using the software program PCORDv6.

Indicator Species Analysis (ISA^87^;), product of the relative abundance and frequency of each family in vent and non-vent zones, was used to identify which families were associated with vent or non-vent zones. Monte Carlo simulations were then used to determine significant indicators by comparing the real Indicator Value (IV) of each family against IVs calculated by randomly assigning observations to zones. ISA was performed in PCORDv6 with 4999 randomized iterations.

A t-test (one way) was used to determine differences in terrain/environmental variables between vent and non-vent zones at Dorado and DSMZ-MBNMS. Variables included northerness, easterness, topographic position, geomorph, average temperature, and substrate (Supplementary Table 7 and Supplementary Table 8). Outliers were assessed using robust fit outlier test with quartiles and K-sigma equal to four in JMP16 on transformed log (x + 1) values. Values were transformed to improve linearity and homogenize variance. One non-vent zone at DSMZ-MBNMS was identified as an outlier. Standard least square multiple regressions were then used to relate variation in family abundance, richness, diversity, and Pielou’s evenness to variables that were found to be significantly different between vent and non-vent zones. These variables were mean temperature and geomorphic setting at Dorado and mean temperature and northerness at DSMZ-MBNMS, excluding the outlier.

The BEST function in PRIMERv6 was used to determine the best match between family composition and environmental (i.e. temperature, geomorphological, and TASSE) variables in vent and non-vent zones. Spearman rank correlations of Bray-Curtis resemblance matrices find the best match between environmental variables and family composition within vent or non-vent zones (rejected if *p* < 1%, as defined by Clarke, 2015)^88^. For each zone type, BEST analysis was run using BIOENV (as opposed to BIOSTEP). This runs all permutations of environmental variables with 499 randomizations. Only those environmental variables that were found to be significantly different between vent and non-vent zones were used in this analysis.

Deep-sea data is often opportunistically obtained, and this study is no exception. Though the data was not uniformly collected, we feel that exploration of these small outcrops resulted in a dataset that provides a comprehensive assessment of environmental conditions and benthic community composition. Over 96 and 57 hours of bottom time imagery were collected at Dorado and DSMZ-MBNMS, respectively. Environmental data was collected every two seconds and high-resolution bathymetry was collected. We used presence in line with deep-sea methods when ROV lighting and footage has limited coverage^89^ and appropriate in machine learning suitability modeling, and restricted family assessment to non-overlapping samples. The authors recognize the challenges this presents in comparing diversity indices. We have elected to use uniform zone size in lieu of transect length, as transects were not planned for community assessment. We also have elected to employ rarefaction curves to correct for sampling size differences. We view this study as a first opportunity to compare communities in the different environments on RFHS and between RFHS themselves.

### Supplementary information

Supplementary Information

Description of Additional Supplementary Files

Supplementary Data 1

Supplementary Data 2

## Data Availability

Acoustic data for Dorado are available NCEI Metadata ID: gov.noaa.ngdc.mgg. multibeam:AT26-09-auv_Multibeam. DSMZ-MBNMS acoustic data are available at Marine Geoscience Data System under Compilations as OctopusGarden_MBARI; DOI: 10.26022/IEDA/331290. JVV images are available at http://4dgeo.whoi.edu/webdata/virtualvan/html/VV-at26-09/index.html. DSMZ-MBNSM NA117 videos are available at: https://www.youtube.com/playlist?list=PL41b0O3MiKnZjVYKiC8zunwc7tGS9L2Az. Chao2 data used to generate the rarefaction curve are for Dorado and DSMZ-MBNMS are available at 10.6084/m9.figshare.25370749.v1. Presence absence community composition and environmental data for Dorado are available at 10.6084/m9.figshare.22350508.v2, and for DSMZ-MBNMS are available at 10.6084/m9.figshare.22350565.v2. Raw data used to calculate average environmental values at Dorado are available at 10.6084/m9.figshare.25345360.v1. Raw environmental data for DSMZ-MBNMS are available at 10.6084/m9.figshare.25343068.v1, except temperature data, which are available at 10.6084/m9.figshare.25345327.v1. Supplementary Data 1 and Supplementary Data 2 are community composition in vent and non-vent zones at Dorado and DSMZ-MBNMS, respectively. Links to Open Source ID Guides: MBARI’s Deep-Sea Guide- http://dsg.mbari.org/dsg/home ; Data: Animal Identification Guide: NOAA Ocean Exploration- https://oceanexplorer.noaa.gov/okeanos/animal_guide/animal_guide.html ; NOAA Ocean Explorer: Living Ocean Gallery- https://oceanexplorer.noaa.gov/gallery/livingocean/livingocean.html (Gallery no longer maintained 2/13/24).
