# Supplementary material for: Natural warming differentiates communities and increases diversity in deep-sea Ridge Flank Hydrothermal Systems
Source: Commun Biol. 2024 Mar 28;7:379. doi: 10.1038/s42003-024-06070-3 (PMC10978836; doi:10.1038/s42003-024-06070-3)
Supplement: Supplementary file 1 — Supplementary Information [file 42003_2024_6070_MOESM1_ESM.pdf]

SUPPLEMENTARY INFORMATION

Supplementary Table 1: Indices for Vent and Non-Vent zones at DSMZ-MBNMS determined in JMP16.

Zone Type	Total species	Total Individuals	Richness	Evenness	Shannon
V	27	232	4.77	0.74	2.45
V	19	162	3.54	0.65	1.92
V	20	116	4.00	0.79	2.37
V	19	107	3.85	0.81	2.37
NV	15	47	3.64	0.84	2.28
NV	13	20	4.01	0.88	2.25
NV	16	91	3.33	0.80	2.21
NV	7	8	2.89	0.98	1.91

Supplementary Table 2: Indices for Vent and Non-Vent zones at Dorado determined in JMP16.

Zone Type	Total species	Total Individuals	Richness	Evenness	Shannon
V	29	81	6.37	0.86	2.88
V	27	79	5.95	0.90	2.96
V	22	37	5.82	0.95	2.95
V	27	44	6.87	0.95	3.12
NV	13	37	3.32	0.85	2.17
NV	5	6	2.23	0.97	1.56
NV	15	32	4.04	0.92	2.49
NV	13	21	3.94	0.91	2.33

Supplementary Table 3: Table of MBES details.

Site (survey month year)	Dorado (December 2013)	DSMZ-MBNMS (February 2022)
Vehicle (affiliation)	AUV Sentry (WHOI)	Dorado mapping AUV (MBARI)
MBES	Reson Seabat 7125	Teledyne Reson T50
Center frequency (kHz)	400	400
Beam Width	1.0° x 0.5°	1.0° x 0.5°
Beams	512	512
Survey Altitude (meters)	50	50
Line Spacing (meters)	50-200	150
Sound Speed	Reson SVP70	SeaBird SBE-49 FastCat CTD
Inertial Navigation System	PHINS	Kearfott SeaDevil
Post processing	MB-System (Tide Correction, sounding editing, navigation, adjustment)	MB-System (Tide Correction, sounding editing, navigation, adjustment)
Product	DTM Generation gridded at 1-meter lateral resolution using MBgrid Beam Footprint Slope algorithm with this plate spline interpolation across 2-meter data gaps	DTM Generation gridded at 1-meter lateral resolution using MBgrid Beam Footprint Slope algorithm with this plate spline interpolation across 2-meter data gaps
Availability	Data available at NCEI Metadata ID: gov.noaa.ngdc.mgg.multibeam:AT26-09-auv_Multibeam MBES generated in Fledermaus by AMH	Caress, D. and J. Paduan, (2023). Near-bottom AUV multibeam bathymetry grid (ESRI ASCII format) from the Octopus Garden hydrothermal spring site. MGDS. doi:10.26022/IEDA/331290

Supplementary Table 4: Descriptions of environmental variables used in the BEST analysis.

Variable	Type	Source	Description
Average Temperature	Quantitative	ROV probe	ROV measured Temperature
Substrate	Numerical	ROV image	Substrate described based off identification in Wheat et al. (2019)
Landform	Numerical	BRESS	Landforms extracted via BRESS using 6 types
Northerness	Quantitative	TASSE toolbox via ArgGIS	Northerness: cosine of angle of slope direction; used to approximate physical processes (radians)
Easterness	Quantitative	TASSE toolbox via ArgGIS	Easterness: sine of angle of slope direction; used to approximate physical processes (radians)
Topographic Position Index (TPI)	Quantitative	TASSE toolbox via ArgGIS	Topographic Position Index- height relative to surrounding pixels; Negative= pits/low/hollow. Positive= crests/highs/peaks (unit-less)

Supplementary Table 5: Numerical values assigned to of BRESS landforms for quantitative analysis.

Value	Landform
1	Flat
2	Footslope
3	Ridge
4	Shoulder
5	Slope
6	Valley

Supplementary Table 6: Numerical values assigned to of Substrate for quantitative analysis.

Value	Substrate
1	Sheet
2	Bare Rock
3	Carbonate
4	Block Sheet/Pillow/Lobate
5	Lobate
6	Pillow
7	Sediment
8	Lobate/Pillow Mix
9	Lobate/Broken/Pillow Mix
10	Sediment/Lobate Contact
11	Lobate/Sheet Mix
12	Lobate/Sediment Mix
13	Lobate/Sheet Contact
14	Sediment/Pillow Mix
15	Other (ROV Arm)

Supplementary Table 7: Environnemental Variable average values in zones at Dorado.

DORADO	n	Northerness	s.d.	Easterness	s.d.	TPI	s.d.	BRESS	s.d.	Average T (°C)	s.d.	Substrate	s.d.
Vent	110	-0.52	0.24	-0.80	0.19	0.001	0.051	4.89	0.46	1.833	0.032	3.75	2.20
Vent	62	-0.71	0.23	-0.59	0.33	-0.010	0.034	5.00	0.00	1.812	0.005	4.24	0.99
Vent	71	-0.68	0.32	-0.23	0.63	0.021	0.088	4.15	0.99	1.818	0.014	4.63	1.11
Vent	50	-0.58	0.18	0.65	0.46	-0.034	0.059	4.68	0.74	1.825	0.020	4.68	1.81
NonVent	29	-0.85	0.18	-0.10	0.50	0.023	0.041	4.21	0.98	1.808	0.005	4.76	1.21
NonVent	12	-0.22	0.44	-0.87	0.17	-0.010	0.055	4.08	1.00	1.806	0.004	4.33	2.23
NonVent	46	0.41	0.37	0.43	0.72	0.087	0.127	3.13	0.50	1.807	0.007	5.75	1.25
NonVent	6	0.05	0.67	-0.77	0.18	0.030	0.030	3.00	0.00	1.806	0.008	4.50	1.22

Supplementary Table 8: Environmental Variable Averages in Zones at DSMZ-MBNMS.

DSMZ-MBNMS	n	Northerness	s.d.	Easterness	s.d.	TPI	s.d.	BRESS	s.d.	Substrate	s.d.	n T-probe	Average T (°C)	s.d.
Vent	29	-0.48	0.71	0.35	0.40	0.0321	0.1484	4.28	0.80	5.86	1.77	1954	2.792	0.950
Vent	15	-0.40	0.18	0.81	0.41	-0.0109	0.1158	5.00	0.00	4.20	0.77	4193	2.594	1.149
Vent	6	-0.44	0.18	0.88	0.10	-0.0467	0.0644	5.00	0.00	10.50	3.56	2213	2.663	1.833
Vent	9	0.09	0.28	0.96	0.05	-0.0063	0.0413	5.00	0.00	4.89	0.33	3434	2.894	1.574
NonVent	8	-0.55	0.16	0.82	0.11	-0.0058	0.0768	5.00	0.00	4.00	0.00	272	1.714	0.012
NonVent	10	-0.94	0.11	0.04	0.33	-0.0586	0.1196	5.00	0.00	10.30	2.21	191	1.705	0.009
NonVent	4	-0.70	0.34	0.56	0.38	0.0321	0.0703	5.00	0.00	4.50	1.00	188	1.679	0.030
NonVent	6	-0.51	0.12	0.85	0.07	0.0002	0.0378	5.00	0.00	6.83	4.67	172	1.704	0.012
