# Supplementary material for: Natural warming differentiates communities and increases diversity in deep-sea Ridge Flank Hydrothermal Systems
Source: Commun Biol. 2024 Mar 28;7:379. doi: 10.1038/s42003-024-06070-3 (PMC10978836; doi:10.1038/s42003-024-06070-3)
Supplement: Supplementary file 2 — Description of Additional Supplementary Files [file 42003_2024_6070_MOESM2_ESM.pdf]

Description of Additional Supplementary Files

File name: Supplementary Data 1
Description: Community Composition in vent and non-vent zones at Dorado Outcrop.

File name: Supplementary Data 2
Description: Community Composition in vent and non-vent zones at DSMZ-MBNMS.
